# Development of Bacterial Biofilms on Artificial Corals in Comparison to Surface-Associated Microbes of Hard Corals

**DOI:** 10.1371/journal.pone.0021195

**Published:** 2011-06-24

**Authors:** Michael John Sweet, Aldo Croquer, John Christopher Bythell

**Affiliations:** 1 School of Biology, Newcastle University, Newcastle upon Tyne, United Kingdom; 2 Departamento de Estudios Ambientales, Universidad Simon Bolivar, Caracas, Venezuela; Université Paris Sud, France

## Abstract

Numerous studies have demonstrated the differences in bacterial communities associated with corals versus those in their surrounding environment. However, these environmental samples often represent vastly different microbial micro-environments with few studies having looked at the settlement and growth of bacteria on surfaces similar to corals. As a result, it is difficult to determine which bacteria are associated specifically with coral tissue surfaces. In this study, early stages of passive settlement from the water column to artificial coral surfaces (formation of a biofilm) were assessed. Changes in bacterial diversity (16S rRNA gene), were studied on artificially created resin nubbins that were modelled from the skeleton of the reef building coral *Acropora muricata*. These models were dip-coated in sterile agar, mounted *in situ* on the reef and followed over time to monitor bacterial community succession. The bacterial community forming the biofilms remained significantly different (R = 0.864 p<0.05) from that of the water column and from the surface mucus layer (SML) of the coral at all times from 30 min to 96 h. The water column was dominated by members of the α-proteobacteria, the developed community on the biofilms dominated by γ-proteobacteria, whereas that within the SML was composed of a more diverse array of groups. Bacterial communities present within the SML do not appear to arise from passive settlement from the water column, but instead appear to have become established through a selection process. This selection process was shown to be dependent on some aspects of the physico-chemical structure of the settlement surface, since agar-coated slides showed distinct communities to coral-shaped surfaces. However, no significant differences were found between different surface coatings, including plain agar and agar enhanced with coral mucus exudates. Therefore future work should consider physico-chemical surface properties as factors governing change in microbial diversity.

## Introduction

Biofilms are complex structures created by microorganisms that attach and grow on available substrates [1]. Most bacteria are capable of forming biofilms and for a large proportion of them this is thought to be their predominant lifestyle [2]. Biofilm formation involves interaction among pioneers and later colonizers, producing temporal shifts in the microbial community structure. Early stages of biofilm formation are not well understood [3], despite its relevance for marine ecological processes such as larval settlement [4], recruitment [3] and the dynamics of microbial communities [5]. Normally, biofilm formation commences with the adsorption of a conditioning film of polysaccharides, proteins, lipids, humic acids, nucleic acids and aromatic amino acids to which the early colonising bacteria subsequently adhere [3]. Growth, reproduction, and death of the primary colonizers modify the characteristics of the substratum, rendering it suitable (or unsuitable) for subsequent colonisation by secondary microorganisms. There is growing evidence suggesting that the early colonizers determine in part the structure of this climax community [5], [6], [7], [8]. Ecological succession via synergistic and/or competitive interactions among these colonists, along with the addition of new accumulating species and/or loss of some previous colonists, will result in a mature, relatively stable climax biofilm community [9].

The surface mucus layer (SML) of corals provides one such surface for the formation of a marine biofilm, as it provides a rich source of carbon and nutrients for settling microbes. Establishment and maintenance of these biofilms could occur in three principal ways depending on the rate of exchange of the SML and the species of coral in question [10]. Microbes could be continually settling or trapped by the mucus but not ultimately forming an established community due to the rapid sloughing off of the layer. If such a transient community existed it might be expected to more closely reflect that of the water column community, although some specificity in settlement processes may exist due, for example, to physico-chemical interactions with the coral SML [11]. Alternatively, a semi-established bacterial community may form in the SML of species of coral that periodically shed their mucus as a tunic (e.g. *Porites* spp.) [12] finally, bacteria might settle and reside in the mucus and/or the coral tissues and become established forming a distinct community from that of the water column. Specific properties of the mucus of different coral species [10] may affect formation of these microbial communities and therefore explain differences in microbial communities of different species [13]. In this final model, although the SML may be continuously or periodically sloughed from the coral surface, either the proportion of the mucus layer replaced and/or the frequency of shedding is insufficient to prevent a stable climax community. Contrary to the first model, the bacterial community structure should in this case remain more stable [14], being determined predominantly by mucus composition [10], [15], and the competitive and antimicrobial properties of the resident bacterial communities [11], [16].

Different studies have shown that corals harbour diverse bacterial communities that differ from the surrounding water environment [14], [17], [18]. The differences in bacterial communities between coral species [19] may be due to differences in the settlement surface offered by each coral species and/or variations in physical and chemical properties of the coral mucus. Corals, with their various microbial environments, (e.g. SML, tissue and skeleton) [20], [21], provide many potential habitats and surface types for a variety of settling bacterial species on a microscopic scale [22]. Different surface properties of such micro-environments are known to affect settlement by influencing cell-cell and cell-surface interactions and thus the formation of the biofilm [23].

Effects of surface type on biofilm development have previously been studied with regard to biofouling [6], [24], [25]. The structure of the settlement surface has been shown to affect the quantity and type of bacteria that can settle, grow, and survive. The physiochemical properties of artificial surfaces that may affect colonization include hydrophobicity, surface free energy, and electrostatic charge [24]. Microorganisms attach more rapidly and build thicker biofilms on hydrophobic and non-polar surfaces, forming an established community that differs strongly to that of the water column. In contrast, communities that form on hydrophilic materials form less actively and result in a bacterial community reflecting the water column. As mucus of the corals is hydrophilic and easily sheared by hydrodynamic forces, bacteria that are incorporated during biofilm formation within the mucus should in theory be similar to those present within the water column, yet this appears not to be the case [14], [20]. Bacterial communities associated with corals differ among and within species [19], suggesting that coral microhabitats and/or their previously established bacterial consortium have the ability to select certain species from the water column and deny settlement of others. In addition to physiochemical properties, antimicrobial activity of the host and/or the resident microbial community have been implicated in this process [17], [26], [27]. The controls on formation of marine biofilms are therefore not well understood. While many studies have examined settlement to flat surfaces, few have employed naturally-shaped objects to determine any hydrodynamic effects on bacterial settlement [24]. We used artificial corals coated in agar to test the different effects of surface shape and chemical composition on the development of a microbial biofilm community over 96 h. We compared these samples to the resident microbial populations associated with the surface mucus layer of a major reef building coral *A. muricata* and that of the surrounding water column. Experiments were repeated during summer and winter to test whether succession was susceptible to seasonality (e.g., differences in water temperature) and samples were collected around the island to assess spatial variability in biofilm formation.

## Results

### Biofilm formation for different substrate types

The artificial coral nubbins were formed from a hard polyurethane resin (Tomps), that was dip-coated in a variety of sterile agar types: plain agar, agar plus mucus, agar plus exudates from a healthy coral, and agar plus exudates from a stressed coral. Although there was a significant difference in C:N ratios between the chosen settling media (ANOVA F = 7.38, p = 0.012) (Table 1), the C:N ratios of all agar types did fall within previous C:N ratios reported for *Acropora* mucus (C:N = 8–14) at the same location [28]. More importantly, there were no significant differences between 16S rRNA gene bacterial assemblages settling on the different agar types (potential food sources) (ANOSIM R = 0.83, p = 0.64) and therefore only plain agar was used for further temporal analysis.

**Table 1:** Percentage carbon and nitrogen and resulting C:N ratio for the four agar types.

Agar type	% N	% C	C : N
Plain Agar	0.63	5.7	9
	0.62	6.12	9.9
	0.62	5.9	9.5
Agar plus mucus	0.32	3.88	12
	0.37	3.83	10.4
	0.33	3.86	11.7
Agar plus healthy coral exudate	0.41	3.9	9.5
	0.44	4.06	9.2
	0.43	4	9.3
Agar plus stressed coral exudate	0.42	3.43	8.2
	0.41	3.99	9.7
	0.41	3.81	9.3

**Figure 1:**
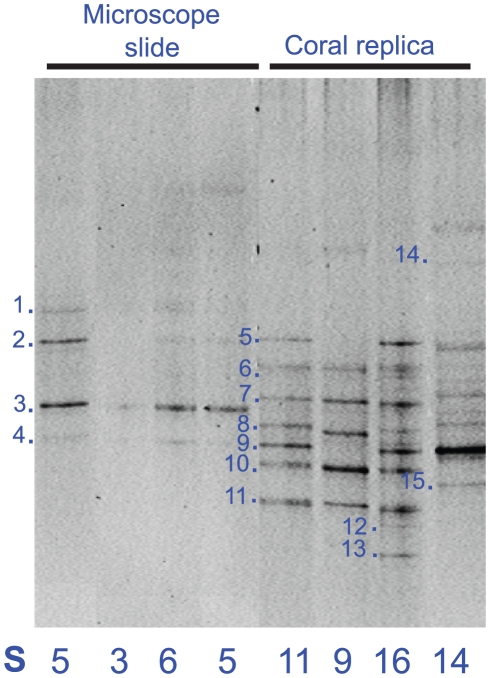
Composite DGGE image showing replicates collected at 4 h of biofilm development on microscopic slides and replica coral nubbins with dominant bands sequenced (**Table 2**), gel-to-gel comparisons were standardised using internally run marker lanes and analysed using BioNumerics software. S =  number of bands visible in DGGE using BioNumerics representing relative diversity.

Microscope slides, dip-coated in the same agar, were deployed on the reef at the same timescales as the artificial coral to compare variations in biofilm development between surface shapes. There was a significant difference between the biofilms that developed on flat surfaces (microscope slides), compared to those on the artificial coral surfaces coated with the same agar (ANOSIM R = 0.84, p = 0.001). A greater diversity of ribotypes were found to settle on the artificial nubbins after 4 h of deployment (S = 9–16; where S =  number of bands visible in DGGE using BioNumerics representing relative diversity), compared to a significantly lower diversity on the smooth surface of the slides (S = 3–6) (Fig 1). Bacteria settling on the artificial coral nubbins included ribotypes similar to *Aeromonas* sp. (AY689043), *Prochlorococcus* sp. (GQ272346), *Shigella* sp. (FJ193359) and *Enterobacter* sp. (FN423410), whilst ribotypes such as *Microbulbifer* sp. (EF674853) and several ribotypes similar to *Pseudoalteromonas* sp. (FM163075, DQ665793 and EU330363), were found to dominate the microscope slide biofilm community (Table 2). Ribotypes of the genus *Pseudoalteromonas* were recorded on both the 4 h developing biofilms on both the slide and the artificial coral nubbin, however no identical ribotypes were found on the two surfaces from dominant bands sequenced from DGGE gels (n = 15) (Fig 1).

**Table 2:** Table showing the dominant 16S rRNA gene ribotypes, explaining the greatest differences/similarities between samples, excised from the DGGE gel.

Band No.	Sample type	Time period	Species ID	Group affiliation	Close relative (% match)
1	Biofilm (agar slide)	4 h	*Pseudoalteromonas sp.*	γ-proteobacteria	FM163075 (99%)
2	Biofilm (agar slide)	4 h	*Pseudoalteromonas sp.*	γ-proteobacteria	DQ665793 (99%)
3	Biofilm (agar slide)	4 h	*Microbulbifer sp.*	γ-proteobacteria	EF674853 (98%)
4	Biofilm (agar slide)	4 h	*Pseudoalteromonas sp*	γ-proteobacteria	EU330363 (97%)
5	Biofilm (agar nubbin)	2/4 h	*Shewanella sp.*	γ-proteobacteria	CP000302 (91%)
6	Biofilm (agar nubbin)	2/4 h	β-proteobacterium	β-proteobacteria	GU257663 (88%)
7	Biofilm (agar nubbin)	2/4 h	*Pseudoalteromonas sp.*	γ-proteobacteria	GQ849227 (98%)
8	Biofilm (agar nubbin)	2/4 h	*Vibrio sp.*	γ-proteobacteria	AB519004 (100%)
9	Biofilm (agar nubbin)	2/4 h	*Klebsiella sp.*	γ-proteobacteria	GQ416635 (90%)
10	Biofilm (agar nubbin)	2/4 h	*Pseudoalteromonas sp.*	γ-proteobacteria	DQ667134 (100%)
11	Biofilm (agar nubbin)	2/4 h	*Aeromonas sp.*	δ-proteobacterium	AY689043 (100%)
12	Biofilm (agar nubbin)	2/4 h	*Prochlorococcus sp.*	Cyanobacteria	GQ272346 (100%)
13	Biofilm (agar nubbin)	2/4 h	*Shigella sp.*	γ-proteobacteria	FJ193359 (91%)
14	Biofilm (agar nubbin)	2/4 h	*Enterobacter sp.*	γ-proteobacteria	FN423410 (100%)
15	Biofilm (agar nubbin)	2/4 h	*Microbulbifer sp.*	γ-proteobacteria	EU837333 (90%)
16	Biofilm (agar nubbin)	24 h	Chloroflexi sp.	Chloroflexi	AB433054 (100%)
17	Biofilm (agar nubbin)	24 h	*Flavobacteriaceae sp.*	Flavobacteria	EF092242 (100%)
18	Biofilm (agar nubbin)	24 h	*Thermus sp.*	Deinococcus-Thermus	DQ989458 (96%)
19	Biofilm (agar nubbin)	24 h	*Pseudoalteromonas sp.*	γ-proteobacteria	FN295786 (100%)
20	Biofilm (agar nubbin)	24 h	γ-proteobacterium	γ-proteobacteria	GU317768 (95%)
21	Biofilm (agar nubbin)	24 h	*Pseudoalteromonas sp*	γ-proteobacteria	GU229650 (91%)
22	Biofilm (agar nubbin)	24 h	*Pseudoalteromonas sp.*	γ-proteobacteria	GU726846 (97%)
23	Biofilm (agar nubbin)	24 h	*Pseudoalteromonas sp.*	γ-proteobacteria	FJ457226 (98%)
24	Biofilm (agar nubbin)	24 h	Cyanobacterium	Cyanobacteria	GU184683 (93%)
25	Biofilm (agar nubbin)	24 h	*Pseudoalteromonas sp*	γ-proteobacteria	FJ237010 (100%)
26	Biofilm (agar nubbin)	24 h	*Pseudoalteromonas sp.*	γ-proteobacteria	GU726846 (100%)
27	Biofilm (agar nubbin)	72/96 h	*Flavobacteria sp.*	Flavobacteria	FN433284 (85%)
28	Biofilm (agar nubbin)	72/96 h	Cyanobacterium	Cyanobacteria	GQ480703 (88%)
29	Biofilm (agar nubbin)	72/96 h	*Glaciecola sp.*	γ-proteobacteria	EU183316 (95%)
30	Biofilm (agar nubbin)	72/96 h	*Planctomycetales sp.*	Planctomycetacia	GU084063 (97%)
31	Biofilm (agar nubbin)	72/96 h	*Aestuariibacter sp.*	Unknown	AB473549 (95%)
32	Biofilm (agar nubbin)	72/96 h	*Klebsiella sp.*	γ-proteobacteria	GQ416635 (95%)
33	Surface Mucus Layer (Coral)	NA	*Chloroflexi sp.*	Chloroflexi	EU909941 (97%)
34	Surface Mucus Layer (Coral)	NA	Cyanobacterium	Cyanobacteria	GQ346809 (100%)
35	Surface Mucus Layer (Coral)	NA	Cyanobacterium	Cyanobacteria	FJ967973 (100%)
36	Surface Mucus Layer (Coral)	NA	Cyanobacterium	Cyanobacteria	FJ946590 (100%)
37	Surface Mucus Layer (Coral)	NA	α-proteobacterium	α-proteobacteria	EF520401 (95%)
38	Surface Mucus Layer (Coral)	NA	δ-proteobacterium	δ-proteobacteria	EF188467 (96%)
39	Surface Mucus Layer (Coral)	NA	*Klebsiella sp.*	γ-proteobacteria	GQ471864 (100%)
40	Surface Mucus Layer (Coral)	NA	*Aeromonas sp.*	δ-proteobacteria	EU919223 (100%)
41	Surface Mucus Layer (Coral)	NA	*Burkholderia sp*	β-proteobacteria	EU876657 (100%)
42	Surface Mucus Layer (Coral)	NA	*Aeromonas sp.*	δ-proteobacteria	EU919223 (100%)
43	Surface Mucus Layer (Coral)	NA	*Klebsiella sp.*	γ-proteobacteria	GQ471869 (100%)
44	Surface Mucus Layer (Coral)	NA	*Streptococcus sp.*	Coccus	DQ001071 (97%)
45	Surface Mucus Layer (Coral)	NA	*Klebsiella sp.*	γ-proteobacteria	GQ471864 (100%)
46	Surface Mucus Layer (Coral)	NA	*Trichococcus sp.*	Coccus	EU919224 (87%)
47	Surface Mucus Layer (Coral)	NA	*Shewanella sp.*	γ-proteobacteria	EU919217 (100%)
48	Surface Mucus Layer (Coral)	NA	*Pseudidiomarina sp.*	γ-proteobacteria	FJ887948 (100%)
49	Water Column (Supply)	NA	*Bacteroidetes sp.*	Bacteroidetes	AM238600 (84%)
50	Water Column (Supply)	NA	Actinobacterium	Actinobacteria	AY632498 (90%)
51	Water Column (Supply)	NA	α-proteobacterium	α-proteobacteria	FJ718457 (96%)
52	Water Column (Supply)	NA	α-proteobacterium	α-proteobacteria	GQ350573 (98%)
53	Water Column (Supply)	NA	α-proteobacterium	α-proteobacteria	GQ204865 (100%)
54	Water Column (Supply)	NA	α-proteobacterium	α-proteobacteria	EF092739 (95%)
55	Water Column (Supply)	NA	*Bacteroidetes sp.*	Bacteroidetes	AB254287 (100%)
56	Water Column (Supply)	NA	α-proteobacterium	α-proteobacteria	FJ620860 (95%)
57	Water Column (Supply)	NA	α-proteobacterium	α-proteobacteria	EU315614 (97%)
58	Water Column (Supply)	NA	*Flavobacteria sp.*	Flavobacteria	EU600663 (100%)
59	Water Column (Supply)	NA	*Bacteroidetes sp.*	Bacteroidetes	EU315425 (96%)
60	Water Column (Supply)	NA	*Flavobacteriales sp.*	Flavobacteria	AB294989 (100%)
61	Water Column (Supply)	NA	α-proteobacterium	α-proteobacteria	FJ532499 (100%)
62	Water Column (Supply)	NA	*Bacteroidetes sp.*	Bacteroidetes	DQ656191 (95%)
63	Water Column (Supply)	NA	γ-proteobacterium	γ-proteobacteria	EU315645 (88%)
64	Water Column (Supply)	NA	γ-proteobacterium	γ-proteobacteria	GQ257639 (82%)

Representatives from each sample types were included; (Biofilm [agar slides], Biofilm [agar coated artificial nubbins], coral mucus and the water column). Close matches (Blast nt), species identification, group affiliation (identified to closest published relatives on GenBank at the time of comparison) are included within the table. All samples were collected from Heron Island reef flat, March 2009.

### Ecological succession of biofilm formation

**Figure 2:**
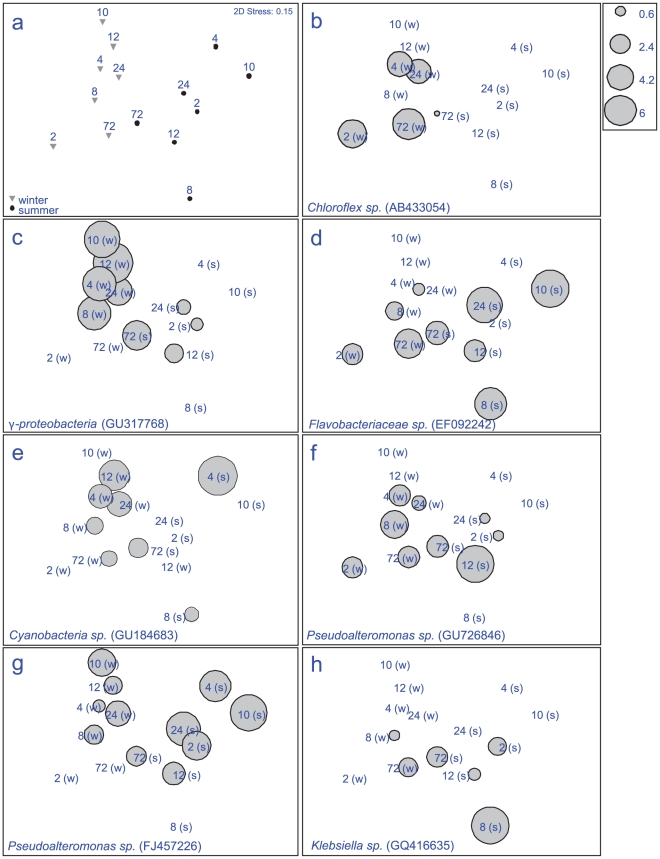
Multidimensional scaling plot (MDS), showing seasonal changes in bacterial communities (16S rRNA gene fingerprints), developing on the biofilm of the replica coral nubbins enriched with agar; (a) average of n = 3 replicates for different time scales of biofilm development for both seasons; summer (s) (March 2009) and winter (w) (August 2008), (b–h) representatives of the sequenced ribotypes responsible for the greatest differences between seasons, Latin name and gen bank sequence ID included. Size of bubble depicts intensity of band/ribotype on DGGE within individual samples.

**Figure 3:**
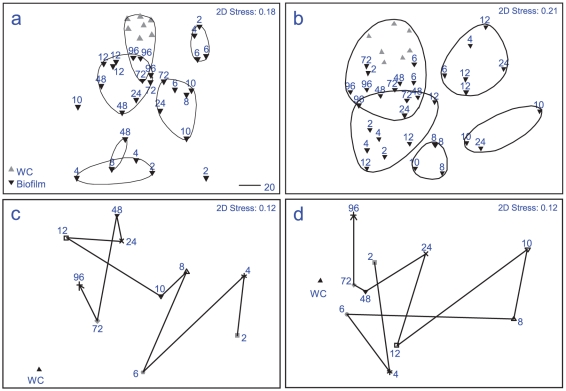
Multidimensional scaling plot (MDS), showing hourly changes in bacterial communities (16S rRNA gene fingerprints), developing on the biofilm of the replica coral nubbins enriched with agar; (a) winter samples (August 2008); (b) summer samples (March 2009). Averages of time periods showing trajectory of similarity between time points, (c) winter and (d) summer. WC  =  water column.

**Figure 4:**
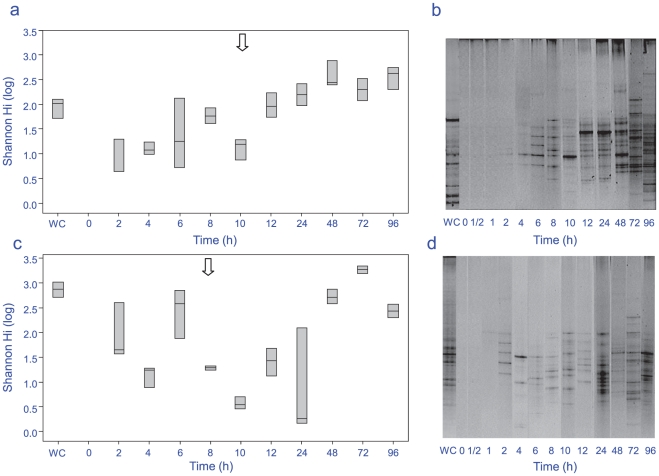
Shannon-Weiner diversity based on DGGE composite (a) Box-plots of the winter season (August 2008), (b) DGGE composite image (winter), (c) box plot of summer season (March 2009), (d) DGGE composite image (summer). Arrow depicts storm event with increased chop (winds above 35 km/h). Average wind speed for other sample periods was below 20 km/h. WC  =  water column.

Significant differences in bacterial assemblages between seasons were observed during biofilm formation (PERMANOVA F = 4.1, p = 0.001), with 22% of the variance between samples explained by season alone. No specific ribotypes occurred exclusively within a single season (Fig 2 a–h), indicating that the significant differences between seasons were due to shifts in dominance of particular ribotypes, not their presence or absence. Ribotypes similar to *Chloroflexi* sp. (AB433054) (Fig 2 b) and a γ-proteobacteria (GU317768) (Fig 2 c) were predominant in winter, where as *Flavobacteriaceae* sp. (EF092242) (Fig 2 d) and a *Pseudoalteromonas* sp. (FJ457226) (Fig 2 g) were found predominantly in summer. Significant shifts in bacterial communities occurred between early bacterial biofilm colonizers (2–12 h), and the later developed community (24–96 h) for both seasons (summer ANOSIM R = 0.442, p = 0.001 and winter ANOSIM R = 0.515, p = 0.001), with a further 23% of the variance being explained by differences between time periods. Large differences (explaining 55% of the variance) between replicates within each individual time period for the first 12 h (Fig 3) indicates a highly dynamic initial settlement period. After 12 h a more stable bacterial community appeared to become established, with only small fluctuations in total diversity afterwards (Fig 3 and 4). During winter, total ribotype diversity (Shannon H^1^), reached that of the adjacent water column after 8 h with a sudden drop at 10 h, potentially brought about by strong weather conditions (i.e. winds above 35 km/h) experienced during this time at the sample site. The diversity recovered subsequently, following a typical asymptotic increase in ribotype diversity through time thereafter (Fig 4 a,b). In summer, there was no such overall pattern in ribotype diversity indicating a more dynamic and less stable biofilm development period during this season (Fig 4 c,d).

The dominant 16S rRNA gene ribotypes seen to be early colonizers between 2–4 h, were absent or undetectable in the later (72–96 h) biofilm (Table 2). A ribotype similar to *Vibrio* sp. (AB519004) present in the 2 h developing biofilm, but absent by 6 h, indicates that this species may be an opportunistic bacterium colonising open spaces, that is later outcompeted by other species such as ribotypes similar to *Flavobacteria* sp. (FN 433284), *Glaciecola* sp. (EU183316), *Klebsiella* sp. (GQ416635), *Aestuariibacter* sp. (AB473549), and a cyanobacterium (GQ480703) (all of which were found after 72 h of biofilm development). Only one ribotype, similar to *Klebsiella* sp. (GQ416635), was consistently detected in both early and late colonising communities. qPCR showed no significant differences between total *Vibrio* DNA dominance of early (185.6±58 fold *Vibrio* DNA template) and late (66±28.3 fold *Vibrio* DNA template) colonizer communities (ANOVA F = 3.43, p = 0.08). However, the mean was 2.8 times larger for early colonizers compared to later stages of biofilm development, and the high variation experienced between replicates might be masking any significant differences.

**Figure 5:**
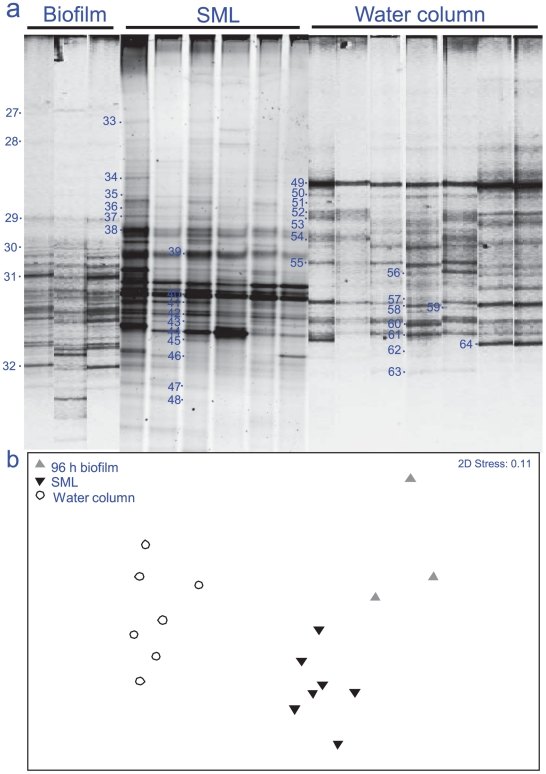
Variation in 16S rRNA gene fingerprints between sample types (Biofilm, SML and water column), for March 2009 (summer); (a) Composite DGGE image standardised for gel-to-gel comparison using BioNumerics, (b) Multidimensional scaling (MDS) plot based on relative band intensity from composite DGGE profile.

During both seasons, there were significant differences between the developing biofilms and the bacterial communities found within the water column (ANOSIM R = 0.907, p = 0.001 for summer, and R = 0.874, p = 0.001 for winter). Pair-wise tests showed significant differences for all time periods of biofilm development (R = 0.864, p<0.05), the only exception being 72 h in the summer season (R = 0.255, p = 0.14). This suggests that the 16S rRNA gene diversity developing on an artificial coral nubbin remains distinct from that of the potential supply from the water column within the timescale studied (Fig 5 a,b).

### Does the SML community represent a particular stage of biofilm development?

**Figure 6:**
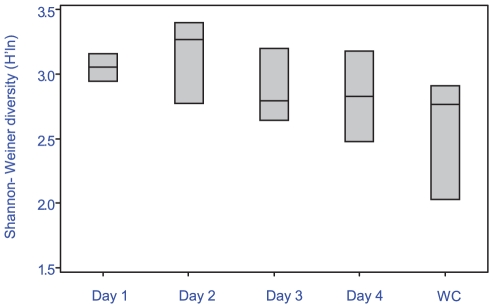
Box-plot showing Shannon-Weiner diversity index of the SML samples of *Acropora muricata* taken over four consecutive days, based on DGGE 16S rRNA gene diversity compared to that of the water column (WC).

Samples from the water column and swabs of the SML were collected alongside the biofilm samples for comparative purposes. There were significant differences between the bacteria which had developed on a 96 h biofilm to those of the coral SML and those present within the water column (ANOSIM, R = 0.5, p = 0.001) (Fig 5 a,b). The water column was dominated by bacteria from the α-proteobacteria group (FJ718457, GQ350573, GQ204865, EF092739, FJ620860, EU315614 and FJ532499), *Flavobacteria* (AB294989 and EU600663), and *Bacteroidetes* (EU315425, AB254287, DQ65619 and AM238600). However, the developing biofilm community after 96 h was dominated by γ-proteobacteria (GQ416635, EU183316, GU726846 and FJ237010) and cyanobacteria (GQ480703 and GU184683). In comparison, the bacteria present in the SML were from a more diverse range of taxa (Table 2, Fig 5 a). Despite the presence of γ-proteobacteria (GQ471864, GQ471869, EU919217 and FJ887948) and cyanobacteria (GQ346809, FJ967973 and FJ946590), there were no exact ribotype matches with those found in the developing biofilm. In addition, the SML of *A. muricata*, showed no significant differences in Shannon diversity based on DGGE 16S rRNA gene diversity (ANOSIM R = 0.569, p = 0.08) over 4 consecutive days of sampling (Fig 6), further indicating that a stable bacterial community, distinct from that in the water column, is present within the SML.

### Spatial variability in biofilm bacterial communities

**Figure 7:**
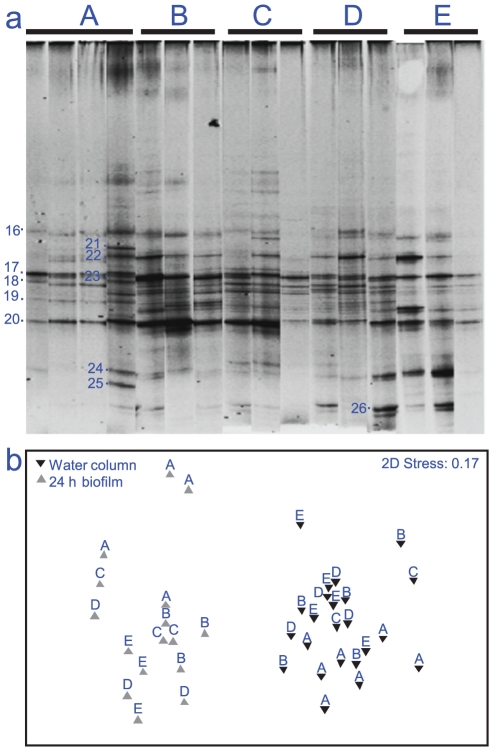
Variation in 16S rRNA gene fingerprints between sample types (spatial samples A–E) for summer season (March 2009); (a) Composite DGGE image standardised for gel-to-gel comparison using BioNumerics, (b) Multidimensional scaling (MDS) plot based on relative band intensity from composite DGGE profile of the biofilm.

**Figure 8:**
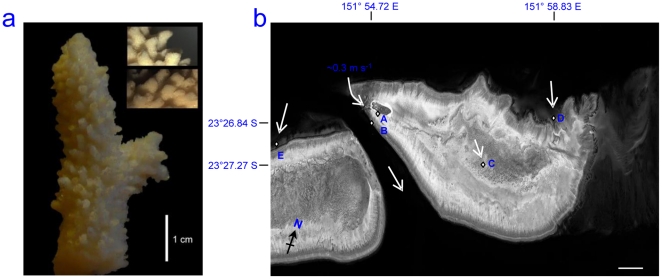
Example of novel methodology utilised in this study and site location; a) Photograph of replica coral nubbins used in experiment with close up sections of the mould (insets), b) Site map showing, Heron Island GBR, Australia (23°27′S, 151°55′E), location of main study site (A) the Reef Flat and those used in spatial sampling; (B) Coral Gardens 23°26.839/151°54.717 (C) Lagoon 23°27.272/151°57.921 (D) 3^rd^/4^th^ Point 23°26.146/151°58.833 (E) Wistari 23°29.081/151°54.015. Arrows depict water current direction at time of sampling with direction and speed noted. Samples were taken on calm days, one hour before high tide, with wave speed W_S_<0.5 m/s and wave heights H_S_<0.5 m. Scale bar  = 1 km.

Spatial variability of 16S rRNA gene diversity was assessed around the Heron Island reef system for both the water column and the developing biofilm, with sites chosen primarily for their differences in benthic structure and predicted water movements. The DGGE profile of bacterial communities developing on artificial nubbins after 24 h showed strong similarities in dominant 16S rRNA gene ribotypes between sites (Fig 7 a). Significant differences were consistently shown between the water column and the 24 h artificial coral nubbin biofilm at all sites (ANOSIM R = 0.874, p = 0.001) (Fig 7 b). Between sites, significant differences were noted for the 24 h artificial coral nubbin biofilm (R = 0.389, p = 0.001), although pairwise tests revealed these differences only between the reef flat and the Wistari reef system (ANOSIM R = 0.667, p = 0.05) (Fig 8b). Similar differences between sites were observed for the water column (ANOSIM R = 0.142 p = 0.05), with pairwise differences between the reef flat and Wistari (ANOSIM R = 0.307 p = 0.001). As such, there were few significant differences between water bodies from deep off-shore, reef, and lagoon waters, as observed for both the water column samples and those of the settling biofilm. Dominant bands were excised from the developing biofilms at the five locations (Fig 7 a), and all samples were dominated by ribotypes similar to *Pseudoalteromonas* sp. (FN295786, GU229650, GU726846, FJ457226 & FJ237010) and a ribotype similar to a *Chloroflexi* sp. (AB433054) (Band 16, Fig 7 a), from the γ proteobacteria and CFB groups respectively.

## Discussion

### Biofilm formation on different substrate types

This study shows that the early-colonizing bacterial communities were strongly affected by the surface structure of the available settlement surface (artificial coral nubbins versus microscope slides) despite being similarly agar-coated. Thomason et al. [24], found significant differences between bacterial communities settling on smooth and textured surfaces in a temperate marine environment, with low dominance found on coarse surfaces and a higher dominance on smoother surfaces. Similarly, in this study we found a greater diversity on the artificial coral nubbins compared to the relatively smooth surface provided by the slides. Surface texture of the artificial coral may provide different opportunity for motile bacteria within the water column to settle, develop, and establish, especially within the branch crevices and individual corallites, where as the slide would offer no such shelter. Developing bacteria on the flat microscope slide may also experience more disturbances from hydrodynamic process such as wave action and current flow. In addition, variation in surface texture may also influence the thickness of the diffusive boundary layer, which would in turn influence the developing bacterial community [29], [30]. Chemical properties of the surface coating had relatively little effect on the developing biofilm as different agar coatings did not result in development of significantly different bacterial biofilm communities. The addition of coral exudates had no significant effect on biofilm formation, suggesting that at these early colonizer stages, factors affecting settlement success, such as surface shape and texture, are more important than factors that may influence growth of the developing community including chemical composition of the coating. Therefore, differences in coral morphology among coral species may play a role (at least in part) in structuring and developing the coral microbiota of specific resident bacterial communities for different coral species [19], [31]. However, autoclaving may have inactivated antimicrobials and other active compounds that may otherwise have led to differential growth.

Bacteria similar to *Aeromonas* sp. (AY689043), *Prochlorococcus* sp. (GQ272346), *Shigella* sp. (FJ193359), *Pseudoalteromonas* sp. (GQ849227) and *Enterobacter* sp. (FN423410), all previously associated with coral tissue and reef systems [32], [33], [34], [35], were the dominant bacteria colonising the artificial coral nubbin. In contrast, on the slides, a ribotype similar to *Microbulbifer* sp. (EF674853) [36] and several species of *Pseudoalteromonas* (EU330363, DQ665793 & FM163075) were dominant, which have not been previously reported in reef systems (and were largely absent from the artificial nubbins). This indicates that the artificial surface more closely represents the natural coral surface and supports the hypothesis that early colonizers may be important to the later development of the coral-associated microbial community.

### Ecological succession of biofilm formation

Succession of bacterial communities in biofilms has been described before [3], [7], [37]. However the exact time frames for settlement of pioneer groups and subsequent recruitment by others is less well understood, which may be due to the majority of studies investigating settlement at >1 d intervals [8], [38]. Some studies have looked at shorter timescales, with Siboni et al. [3] reporting colonisation of bacteria on surfaces after 2 h in marine environments. In the present study, samples taken at 30 min and 1 h after redeployment failed to show any bacterial community using the technique utilised(DGGE). Several studies have shown pioneer communities (developing between 0–9 h), consisting of mainly γ-proteobacteria (*Pseudomonas*, *Actinetobacteria* and *Alteromonas*), with a subsequently more stable biofilm developing after 24 h and dominated by α-proteobacteria in varying marine environments [5], [6], [8], [38]. Our results suggest that γ-proteobacteria are the dominant group of early settlers (<24 h), however the later shift to α-proteobacteria seen by these previous studies [6], [8], [39] was not detected in this case. In addition, in this study we did not see an asymptotic maximum diversity reached within 96 h, compared to the maximum reached within 36 h reported in the study by Lee et al. [5]. The bacterial community developing on the biofilm in this case at least, may not have reached a stable equilibrium.

Seasonality undoubtedly has an important influence on the formation of biofilms, as seen by the significant differences between samples from summer and winter; a result similarly reflected in other systems [40]. Ceh et al. [41], suggest seasonal changes are the primary factor driving the microbial consortium in coral-bacterial associations, rather than species [19] and spatial separation [42], [43]. However, seasonal changes include several different factors that can affect such microbial biofilm development. Biotic factors, such as the chemical composition of the coral SML [44], [45], exudation of other substances such as antimicrobials [16], [46], [47], and the activity of grazers on the biofilms [48] would undoubtedly play an important role in microbial community development [49], [50]. Furthermore, abiotic factors, such as temperature [50], [51], wave action [52], light conditions [53], and seawater nutrient levels [54] would also affect settlement and growth.

### Does the SML community represent a particular stage of biofilm development?

Previous studies have shown clear differences between free-living bacteria and those developing on biofilms [1], [5], [8], [40], [55], although initial biofilm formation is from the attachment of specific groups of these free-living bacteria sourced originally within the water column [8]. In this study, the bacterial diversity of the developing biofilms remained clearly different from that of the potential supply (the water column), even in the earliest detectable stages of development (∼2 h). The water column was dominated by α-proteobacteria, Flavobacteria and Bacteroidetes, compared to that of the developing community on the biofilms being largely γ-proteobacteria. We hypothesised that the bacterial community would initially be more similar to the water column, driven by passive, non-selective settlement, yet would become progressively more dissimilar as selection and growth of the biofilm community occurred. However, our results suggest that the developing biofilm bacteria must be recruited from the onset from less abundant populations within the water column, through selective processes or via transmission of bacteria by direct contact with other surfaces (e.g., sediment transported via wave action). These bacteria may then undergo rapid growth (with the availability of additional food sources) and therefore become the dominant detectable group on the biofilm. Due to limitations in the resolution of the DGGE technique, rare populations in the water column are not readily detected, making it difficult to correlate potential fluctuations in the water column of these less dominant bacterial species present within the community with those in the developing biofilms, as seen in this and previous studies [5], [40], [49], [56].

The difference in developing bacterial communities on any surface can be explained in part by the fact that some marine macro-organisms (like corals) combat microbial fouling by producing compounds that inhibit bacterial growth or attachment [44], [46], [47], where as others rely on microbial production of defence compounds [11], [12], [21]. In addition, even on inert objects like the artificial corals used in this study, commensal relationships (bacteria-bacteria interactions) can play an important role in determining the spatial distribution of microbial populations within a developing biofilm [11]. Bacteria such as *Alteromonadales*, and in particular *Pseudoalteromonas* sp., like those found predominantly as early colonizers in this study, have previously been shown to be highly antagonistic both at normal and elevated temperatures, and will actively inhibit other species from settling or establishing [2], [16], [57], [58]. *Pseudoalteromonas* strains can therefore predominate over other bacterial strains such as potentially pathogenic *Vibrio* sp. [2], producing a variety of biologically active extracellular compounds, including antibacterial agents that ultimately lead to antifouling effects [59], [60]. Interestingly, some γ-proteobacetria have also been shown to be specific with their antagonistic behaviour, inhibiting only other α- proteobacteria from growing [16].

Rypien et al. [16] found that pathogenic *Vibrios*, in particular *V. shiloi* and *V. coralliilyticus,* are usually inhibited by other coral-associated bacteria found in healthy coral samples. During periods of stress, these natural inhibitors are reduced in number and less able to inhibit the potentially pathogenic *Vibrios*, allowing these pathogenic bacteria to become overwhelming and cause disease [16]. Although qPCR showed no significant difference in total *Vibrio* numbers from early to late colonizers, one *Vibrio* sp. (AB519004) was shown to be an early colonizer and was absent in later stages of the biofilm development. This indicates that at least this particular species was outcompeted by more dominant types such as ribotypes similar to *Flavobacteria* sp. (FN433284), *Glaciecola* sp. (EU183316), and *Aestuariibacter* sp. (AB473549), along with a cyanobacterium (GQ480703). The only ribotype found consistently between the biofilms and the SML was a ribotype similar to *Klebsiella* sp. (GQ416635).

### Spatial variability in biofilm bacterial communities

Although there were few significant differences between sampling sites (either in the water column or the developing biofilm), the samples between which significant differences did occur (i.e., the reef flat and the Wistari reef system) exhibited consistent differences in both the water column and the developing biofilms. This repeating pattern indicates rapid benthic-pelagic coupling in the microbial communities, although as of yet it is impossible to infer whether the later developing community was controlled by initial colonizers [2], [5], [6] or alternatively by continual settlement from the water column [3].

In conclusion, the developing bacterial community found on biofilms remains distinct from that of the potential supply (i.e. the water column), and those bacterial communities present within the SML. Surface structure, but not material composition, significantly affects the initial bacterial community assemblages, therefore, future work looking at biofilms should carefully consider surface properties as a factor governing change. The seasonal differences reported here indicate that biofilm development varies from summer to winter months, reflected but not consistent with, the difference in bacterial communities found within the water column between seasons [61].

## Materials and Methods

### Experimental design

In order to assess the temporal dynamics of the microbial community settling and developing on the coral surface, an artificial surface was created that resembled the coral surface in both structure and food source availability. Artificial coral nubbins were modelled after the scleractinian coral *Acropora muricata* ( = *A. formosa*) by producing a mould of silicone rubber. The artificial nubbins were formed from a hard polyurethane resin (Tomps), and had the same size and identical structure (to the microscopic level), allowing for standardised replication (Fig 8a). All models were bathed in filtered seawater (FSW) (0.22 µm), for 24 h prior to use, further washed in fresh FSW three times and left under a high energy ultra violet (UV) light overnight to sterilise the nubbins [62]. This process was used to remove any potential chemical and/or bacterial contaminants, which may have occurred during the production process or transportation to the field site. Each artificial nubbin was dip-coated twice in sterile unaltered agar (Difco; 1.5% w/v), giving an even coat of between 0.5–1 mm thickness, resembling a food source and thickness (0.5–0.8 mm) naturally provided by the SML of corals [28], [63]. Although the nutritional and biophysical properties of coral SML could not be reproduced, we aimed to test the effects of different growth media (see below), in order to explain the effects of differential settlement versus differential growth on the developing community. The study was conducted at Heron Island, Great Barrier Reef, Australia (Fig 8b), over two years, encompassing both a summer (March 2009) and winter (August 2008) season. The average sea surface temperatures during these months at the site ranged from 26–28°C during the summer sampling period and 20–22°C during the winter. The artificial coral nubbins (n = 36) were placed on the reef flat (Fig 8b A), using a push mount system [20]. Subsequently, the nubbins were sampled over a time series (30 min, 1 h, 2 h, 4 h, 6 h, 8 h, 10 h, 12 h, 24 h, 48 h (2 days), 72 h (3 days), and 96 h (4 days), which allowed monitoring of the natural development and succession of bacteria over time. At each time period replicates (n = 3) of the artificial coral samples were collected in sterile 50 ml falcon tubes, which were placed in an autoclaved bag on return to the laboratory. The agar was then airbrushed off and scraped into a sterile micro centrifuge sample tube with absolute ethanol using sterile scalpel blades, after which the agar was stored and kept at −20°C until extracted.

To assess the effects of growth media on the developing bacterial community we employed four variations in marine agar types. The four agar types were made up as per the manufacturer guidelines (Difco) using 0.22 µm filtered FSW collected on site: 1) plain agar, 2) agar plus mucus from the coral *A. muricata* collected *in situ* (five nubbins of *A. muricata* were exposed and inverted upside down with the resulting mucus collected (100 ml in total) into a sterile container [11], which was later made up to a total 500 ml of agar before autoclaving), 3) agar plus healthy coral exudates (where a ∼15 cm diameter colony of *A. muricata* had been bathed in 5 l of water for 24 h under constant seawater flow and 26°C) filtered through a 0.22 µm polycarbonate filter and made up as per manufacturers guidelines, and 4) agar plus stressed coral exudates (where a similar sized coral colony was exposed to extreme levels of sunlight in a shallow tank for 24 h). Replicates (n = 4) of each agar type were sampled for each time period. Samples of each agar were taken at time of preparation, freeze-dried and crushed, then 10 mg were placed in 5×9 mm tin capsules (Costech Analytical Technologies) and analysed for C and N composition to compare between the different types (School of Chemistry, Newcastle University). In order to compare the developing bacterial communities on different surface structures, sterile microscope slides (n = 36) were dip-coated in plain agar, (no modifications), and mounted vertically. These were then deployed at the same time intervals as the artificial coral nubbins to allow for comparisons between biofilm development on flat surfaces and those that develop on textured surfaces (artificial coral nubbin). Agar from microslides were processed and stored as above.

To assess spatial variation around the island reef system, samples of the artificial coral nubbins coated in plain agar (as per manufacturers guidelines) were set out at five locations around Heron Island (Fig 8b, A–E) for 24 h periods. These samples were collected at high tide to estimate spatial variability in bacterial biofilm diversity and composition. The sites were chosen at time of sampling, as they were expected to show variation in their bacterial diversity due to differences in the benthos (e.g. sandy lagoon site C compared to reef crest site B) and known oceanographic patterns around the island [61]. The spatial sample artificial nubbins and subsequent water sampling were sampled during the summer season only.

In order to assess if the SML of reef building corals represented a particular stage of biofilm development and if the water column was the supply of these developing microbes, water column samples (n = 36) were taken at the same time as each of the biofilm samples, and coral mucus swabs (summer season only) (n = 4), were also collected. For the water samples, 1 l of water ∼5 cm above the coral colony was continuously sampled for a period of 1 h, onto 0.22 µm Sterivex filters, using a Masterflex pump [61]. For mucus samples, approximately 20 cm^2^ of the branch tip of colonies of *A. muricata* was swabbed using sterile cotton buds and immediately placed in sterile universal micro centrifuge tubes with ethanol [14], [20]. All samples were collected in sterile micro centrifuge tubes at time of sampling, allowing no contact with the air during collection and transport back to the laboratory and stored at −20°C until processed.

### Bacterial 16S rRNA gene diversity, DNA extraction, amplification and DGGE analysis

DNA was extracted from all samples using QIAGEN DNeasy Blood and Tissue kits with an added step to concentrate the lysate using vacuum centrifugation for 2 h at 20°C. Bacterial 16S rRNA genes were amplified using standard prokaryotic (357F) **(** 5′-CCTACGGGAGGCAGCAG-3′) and (518R) (5′-ATTACCGCGGCTGCTGG-3′) primers. These primers were chosen over more traditional ones as they have been recently shown [64] to more comprehensively amplify marine bacteria compared to inadequacies and mismatches caused by those such as 907r (pC) [14], [64], [65]. The GC – rich sequence 5′ – CGC CCG CCG CGC GCG GCG GGC GGG GCG GGG GCA GCA CGG GGG G-3′ was incorporated in the forward primer 357 at its 5′ end to prevent complete disassociation of the DNA fragments during DGGE. Thirty PCR cycles were performed at 94°C for 30 seconds, 53°C for 30 seconds and 72°C for 1 min and a final extension at 72°C for 10 min [64]. A 30 µl PCR reaction was used containing 1.5 mM MgCl_2_, 0.2 mM dNTP (PROMEGA), bovine serum albumin (BSA, 400 ng µl^−1^), 0.5 mM of each primer, 2.5 U of Taq DNA polymerase (QBiogene), incubation buffer, and 20 ng of template DNA [3]. All reactions were performed using a Hybraid PCR Express thermal cycler. PCR products were verified by agarose gel electrophoresis (1.6% weight/volume agarose) with Ethidium Bromide staining and visualized using a UV transilluminator.

DGGE was performed using the D-Code universal mutation detection system (Bio-Rad). PCR products were resolved on 10% (w/v) polyacrylamide gels that contained a 30–60% denaturant gradient for 13 h at 60°C and a constant voltage of 50 V. Gels were stained with a concentrated solution of 9 µl SYBR® Gold (Sigma) in 50 µl of 1X TAE poured directly onto the gel surface, covered and left in the dark for 20 min then further washed in 500 ml 1X TAE for 30 min and visualized using a UV transilluminator. Dominant bands of interest (those which explained the greatest differences/similarities between samples) were excised from DGGE gels for the summer season only, left overnight in Sigma molecular grade water, vacuum centrifuged, re-amplified with primers 357F and 518R [61], labelled using Big Dye (Applied Biosystems) transformation sequence kit, and sent to Genevision (Newcastle University, UK) for sequencing. Bacterial operational taxonomic units (OTUs) [14] were defined from DGGE band-matching analysis using BioNumerics 3.5 (Applied Maths BVBA). Standard internal marker lanes were used to allow for gel-to-gel comparisons. Tolerance and optimisation for band-matching was set at 1%.

Quantitative PCR (qPCR) was conducted on an Engine Opticon® 2 system in order to test whether *Vibrio* sp. abundance (a genus of bacteria, known to contain potential opportunistic pathogens implemented in coral diseases [66], [67]) changed between biofilm development times (n = 20 randomly chosen samples): 10 from both the early colonizers (classed as 2–12 h) and later colonizers (established communities, classed as 24–96 h). For this, *Vibrio*-specific primers were used (567F, 5′-GGCGTAAAGCGCATGCAGGT-3′; 680R, 5′-GAAATTCTACCCCCCTCTACAG-3′ [68]), that have previously been shown to be highly targeted towards *Vibrios*, matching 42 out of 43 sequences of *Vibrio* type strains in the RDP database [68]. qPCR reaction mixtures totalled 25 µl and consisted of 12.5 µl of 2X Quantitect® SYBR® Green 1 supermix (Qiagen), 1.25 µl each of 0.5 mM forward and reverse primers, 50 ng DNA and 9.5 µl Sigma molecular grade water. Each set of samples included a negative control, in which water was substituted for the DNA sample. qPCR was performed with an initial activation step of 15 min at 95°C, followed by 39 cycles (94°C for 15 s, 58°C for 30 s, primer annealing at 58°C for 30 s). The fluorescent product was detected after each extension. Following amplification, melting temperature analysis of PCR products was performed to determine the specificity of the PCR. The melting curves were obtained by slow heating at 0.5°C s^−1^ increments from 50 to 90°C, with continuous fluorescence recording.

### Statistical analysis

Matrices of Bray-Curtis similarities were generated using band intensity data (where 0 =  absence), from the DGGE analysis, using marker lanes for between-gel comparisons. An analysis of similarities (ANOSIM, [69]), was performed to compare changes in bacterial community structure that developed onto the different types of agar. Likewise, bacterial communities which developed onto artificial coral models and slides were compared with an ANOSIM test. Temporal changes in bacterial assemblages were also evaluated with a two-way permutation analysis of variance (PERMANOVA), and multi dimensional scaling (MDS), based on Bray-Curtis similarities. A one-way analysis of similarity (ANOSIM), was performed separately for summer and winter data sets. A similarity profile analysis (SIMPER), was performed in order to determine the ribotypes that contributed most to the observed patterns. Average similarities (centroids), of bacterial communities were estimated from replicates corresponding to each time point. These centroids were used to produce new MDS plots showing the temporal trajectory (i.e., succession) of bacterial assemblages from initial settlement up to 96 h. Shannon-Weiner diversity indices were used to compare temporal samples for each season. The 16S rRNA gene diversity settling on the artificial coral for 96 h biofilm development and those of the coral SML were compared with those present within the water column using band intensity data and an MDS plot. qPCR calculations were based on relative DNA concentration (ΔCt) of *Vibrios* based on lowest detected concentration (Ct). Fold differences in *Vibrio* DNA template were calculated assuming 2-fold PCR reaction efficiency (2^ΔC(t)^). One way ANOVA (minitab) was used to compare between settler communities.
